# Association Between Maternal Gestational Diabetes, Cord Blood DNA Methylation, and Offspring Neurodevelopment

**DOI:** 10.3390/ijms27083571

**Published:** 2026-04-16

**Authors:** Nieves Luisa González-González, Marina Armas-González, Enrique González-Dávila, José Ramón Castro-Conde, Candelaria González-Campo, Carlos Flores, José Miguel Lorenzo-Salazar, Rafaela González-Montelongo, Adrián Muñoz-Barrera, Erika Padrón-Pérez, Laura Tascón-Padrón, Olivia Orribo-Morales

**Affiliations:** 1 Department of Obstetrics and Gynecology, Sección de Medicina, Facultad de Ciencias de la Salud, University of La Laguna (ULL), 38320 La Laguna, Spainlalytfe@gmail.com (C.G.-C.); erikapadronperez@gmail.com (E.P.-P.); oorribomorales@gmail.com (O.O.-M.); 2 Department of Child and Adolescent Psychiatry, Hospital General Universitario Gregorio Marañón, 28007 Madrid, Spain; 3 Department of Mathematics, Statistics and Operations Research, Instituto de Matemáticas y Aplicaciones (IMAULL), University of La Laguna (ULL), 38320 La Laguna, Spain; egonzale@ull.edu.es; 4 Department of Pediatrics, Canary Islands University Hospital, 38320 La Laguna, Spain; 5 Research Unit, Hospital Universitario Nuestra Señora de Candelaria, Instituto de Investigación Sanitaria de Canarias (IISC), 38010 Santa Cruz de Tenerife, Spain; cflores@ull.edu.es; 6 CIBER de Enfermedades Respiratorias (CIBERES), Instituto de Salud Carlos III, 28029 Madrid, Spain; 7 Genomics Division, Instituto Tecnológico y de Energías Renovables (ITER), 38600 Santa Cruz de Tenerife, Spain; jlorenzo@iter.es (J.M.L.-S.); amunoz@iter.es (A.M.-B.); 8 Department of Obstetrics and Gynecology, Canary Islands University Hospital, 38320 La Laguna, Spain; 9 Department of Obstetrics and Prenatal Medicine, University Hospital Bonn, 53127 Bonn, Germany; l.tasconpadron@gmail.com

**Keywords:** gestational diabetes mellitus (GDM), DNA methylation, epigenetic, neurodevelopment, Bayley-III, whole-genome bisulfite sequencing (WGBS), fetal programming, cord blood, genes

## Abstract

The link between neurodevelopment in infants exposed to maternal gestational diabetes mellitus (GDM) and fetal DNA methylation remains unexplored. We conducted this hypothesis-generating study to investigate the association between fetal DNA methylation and neurodevelopmental outcomes in children of mothers with GDM. We carried out a prospective, observational pilot cohort study comparing infants exposed to maternal GDM with an unexposed control group. Umbilical cord blood DNA methylation was assessed using targeted methylome sequencing covering 3.34 million CpG sites. Infant neurodevelopment was evaluated at age two years using the Bayley-III Scales. Bioinformatics processing identified differentially methylated regions (DMRs), followed by multiple enrichment analyses of DMR-associated genes and partial correlation analyses. Multi-dimensional enrichment analysis of the 1053 identified DMR-associated genes revealed a significant convergence of pathways related to neurogenesis, synaptic components, and axonal guidance. Infants born to mothers with GDM exhibited lower scores in cognitive, language, and motor domains, which were associated with identifiable DNA methylation signatures at birth. Significant correlations were observed in genes essential for brain scaffolding and synaptic circuitry, most notably *WNT4*, the *PCDHG* alpha/beta clusters, and *PALM*. Additionally, methylation patterns in *FOXF2* and *CHFR* suggest a potential impact on blood–brain barrier integrity, while associations with *FSTL3* and *H6PD* highlight a systemic metabolic ‘cross-talk’ influencing neurodevelopment. Although these pilot findings are hypothesis-generating and require further functional validation, this study provides pioneering evidence that neurodevelopmental alterations in the offspring of mothers with GDM are potentially associated with intrauterine epigenetic modifications detectable at birth.

## 1. Introduction

Gestational diabetes mellitus (GDM) is defined as glucose intolerance first recognized during pregnancy, with a global prevalence estimated at approximately 14% [1]. The incidence of GDM is rising worldwide, paralleling increases in maternal age and obesity, and it represents one of the most common metabolic complications of pregnancy [2]. The clinical significance of GDM stems from its strong association with immediate obstetric and neonatal morbidity as well as its capacity to alter the long-term physiological trajectories of both the mother and the offspring [3].

Maternal diabetes, including GDM, has been consistently linked to increased risks of neurodevelopmental disorders in offspring, such as intellectual disability, learning disorders, attention-deficit hyperactivity disorder or autism spectrum disorder [4,5,6]. The severity of these outcomes is influenced by the timing and degree of maternal hyperglycemia [7].

Epigenetics is defined as the study of heritable changes in gene expression that occur without altering the underlying DNA sequence. These modifications regulate the “on/off” state of genes and mediate tissue-specific gene expression and cellular differentiation, driving offspring developmental programming in response to the interplay between genetic predisposition and the intrauterine environment, including maternal metabolic health. Among the various epigenetic mechanisms, DNA methylation—characterized by the addition of a methyl group (CH_3_) to the C5 position of the cytosine ring at CpG sites (cytosine–guanine dinucleotides)—remains the most widely documented and extensively studied [8].

There is growing evidence that maternal GDM induces DNA methylation changes in offspring, which are associated with an increased risk of obesity, type 2 diabetes, and insulin resistance later in life. These epigenetic modifications are detectable at birth in umbilical cord blood [9,10,11]. However, to the best of our knowledge, no research has yet evaluated the potential links between epigenetic modifications conditioned by in utero exposure to maternal GDM and neurodevelopmental alterations in human offspring. The only existing report exploring this possibility was conducted in fetuses of diabetic rats and focused exclusively on a single gene [12].

We hypothesize that the intrauterine environment in GDM-complicated pregnancies induces specific epigenetic modifications in the offspring that are detectable at birth and functionally associated with the children’s neurodevelopmental trajectories observed at 24 months of age.

The objective of this study was to determine whether newborns exposed in utero to maternal GDM exhibit DNA methylation changes in cord blood associated with their subsequent neurodevelopment, measured by the Bayley-III Scales. Given the limited evidence regarding this specific link, we conducted this investigation as a hypothesis-generating pilot study.

## 2. Results

A detailed comparison of maternal profiles and perinatal outcomes is presented in Table 1. The mean HbA1c values were 6.1% ± 0.8, 5.7% ± 0.7 and 5.7% ± 0.8 in the first, second and, third trimesters, respectively. Non-obstetric complications or perinatal complications were recorded.

### 2.1. Genome-Wide Methylation Analysis

Of the 1,857,654 individual CpG sites analyzed per sample, 1345 were identified as differentially methylated (DM) (methylation difference > 15%, q < 0.01). Among these, 825 (61.3%) exhibited hypomethylation, with mean differences ranging from −35.0% to −15.0%, while 520 (38.7%) were hypermethylated, with differences ranging from 15.0% to 36.6%. In addition, 1053 DMR-associated genes were identified (methylation difference > 1%, q < 0.01).

### 2.2. Gene-Set Enrichment Analysis

Gene Ontology (GO). Functional enrichment analysis of the 1053 DMR-associated genes identified was performed by inputting them into the Gene Ontology platform [13] with a background set of 20,580 uniquely mapped genes. Of these, 885 genes were unequivocally mapped to the human genome (192 were unmapped, and 27 showed multiple-mapping information).

Among the top fifteen enriched pathways (Supplementary Table S1), three were directly linked to neurodevelopmental processes: vocal learning, with this category encompassing imitative and observational learning, memory, and behavior; walking behavior; and axon guidance, including sub-terms like neuron projection morphogenesis and axonogenesis. The detailed results for GO enrichment analysis are presented in Table 2.

Analysis using the COMPARTMENTS database [14] identified significant enrichment in cellular components essential for neuronal architecture and signaling. The most prominent pathways included main axon, axon initial segment, and neuron projection (Table 3 and Figure S1, Supplementary Materials).

Finally, the SynGO enrichment analysis [14] revealed a significant convergence of DMR-associated genes onto terms critical for synaptic structure and function. The identified pathways exhibit distinct clustering, particularly within the postsynaptic density and neurotransmitter receptor activity branches, suggesting a coordinated epigenetic impact on the developing synapse (Table 4 and Figure S2, Supplementary Materials).

### 2.3. Neurological Assessment of Infants

**Figure 1:**
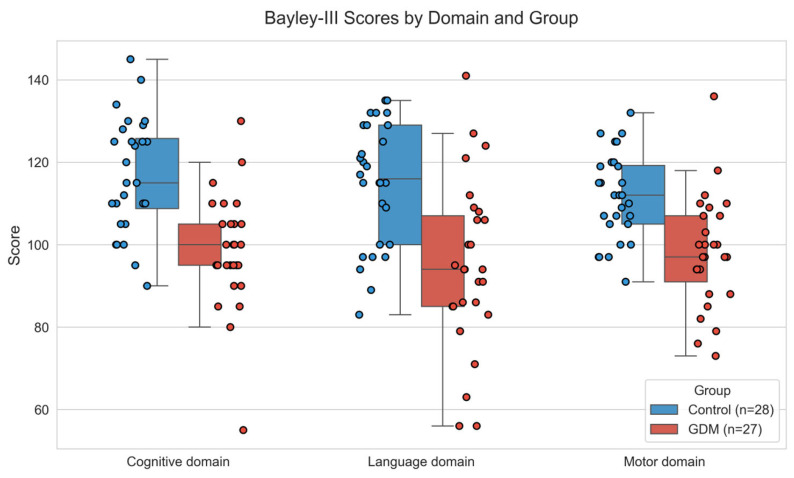
Boxplots showing cognitive, language, and motor domain scores on the Bayley-III Scales for infants exposed to maternal GDM and those born to non-GDM mothers (controls). Differences between the GDM and Control groups were assessed using Student’s *t*-tests for each domain, with all domains showing a significant level of *p* < 0.001. The points represent individual scores for each participant.

Neurological follow-up of infants at two years of age was compromised in five cases due to parental non-adherence. Ultimately, a final cohort comprising 27 infants of mothers with GDM (IGDM) and 28 control infants were examined using the Bayley-III Scales of Neurodevelopment [15]. At two years of age, IGDM showed significantly lower scores than controls in cognitive (*p* < 0.001), language (*p* < 0.001), and motor (*p* < 0.001) domains on the Bayley-III Scales (Table 1 and Figure 1).

### 2.4. Correlations Between Methylation Levels in DMRs and Bayley-III Scores

Partial correlation analysis, controlling for the potential confounding effect of gestational age, revealed significant associations between 28 DMR-associated genes and subsequent Bayley-III scores.

Methylation values in DMRs associated with *FOXF2* and *WNT4* demonstrated significant correlations across all developmental domains of the Bayley-III Scales. Methylation levels in DMRs associated with *TBCD*, *CHFR* and *LOC101928978* were significantly correlated with two domains (cognitive and language). The remaining 23 DMR-associated methylation sites correlated with scores in only a single domain.

Cognitive Domain: Scores were significantly correlated with methylation levels at the DMR-associated *PCDHGA1-8*, *A11*, *B1-4*, and *B8P* clusters as well as within the *C1QTNF6*, *FSTL3/PALM*, and *H6PD* genes.

Language Domain: Methylation levels showed significant correlations with performance in loci overlapping with *NLRC5*, *C1QTNF8*, *PPP2R5C*, *KCNT1*, *SLC37A1/PDE9A*, *CSNK1D* and *TP73-AS1.*

Motor Domain: Additionally, significant correlations were observed between motor domain scores and methylation levels in the following DMR-associated genes: *ENPP7P13*, *GALNT9*, *TMEM132D*, *MEST*, *BRF1/BTBD6*, *FMO2*, *SCGB1B2P*, *LINC01626*, *HOXB3*, and *GNAS.*

**Figure 2:**
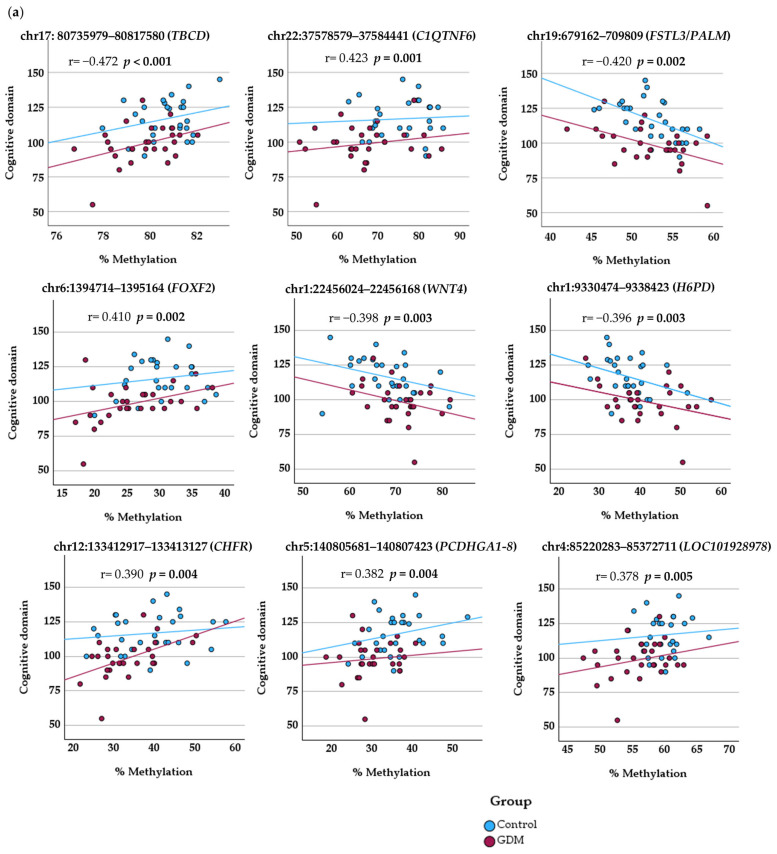

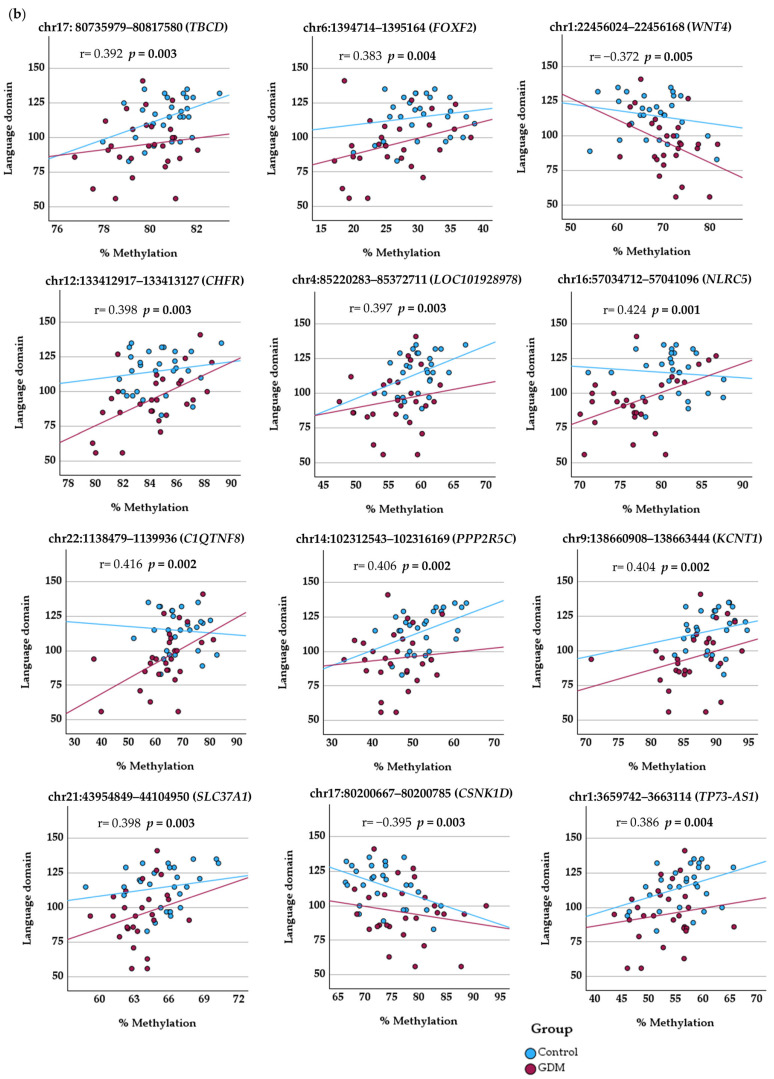

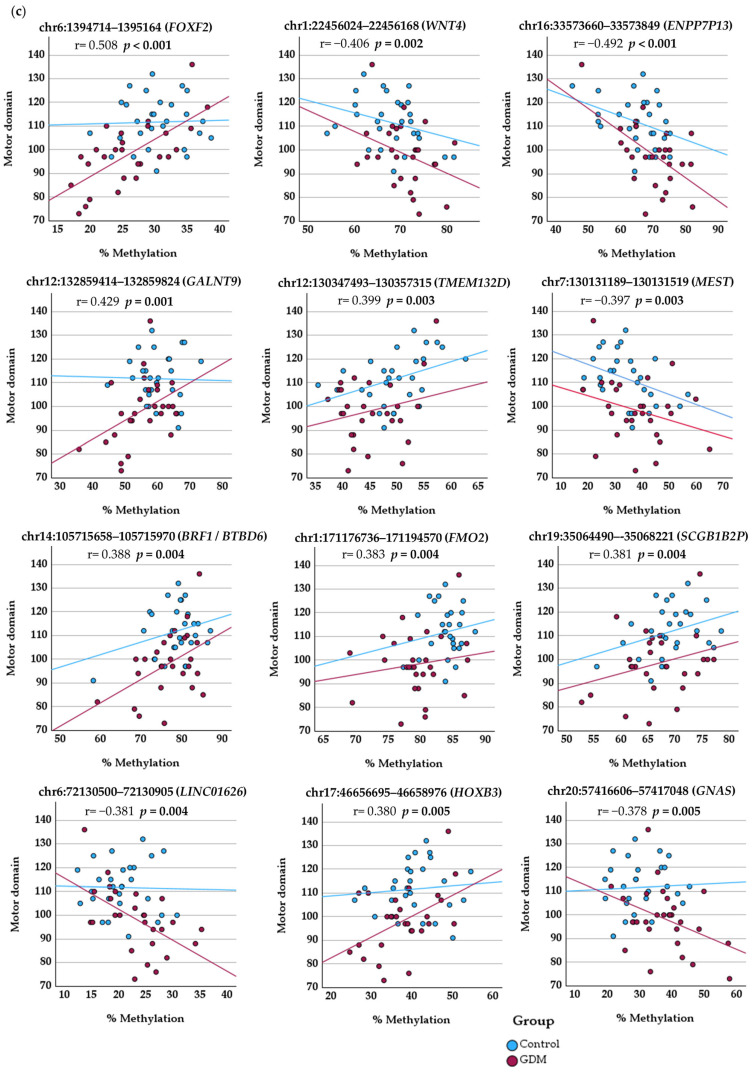
Correlation of Bayley-III neurodevelopmental scores with DNA methylation levels (%) at differentially methylated loci. Scatter plots showing partial correlations between DNA methylation and Bayley-III scores, adjusted for gestational age. (**a**) Cognitive domain; (**b**) language domain; and (**c**) motor domain. Solid lines represent the linear regression for each group (Control and GDM).

Table 5 and Figure 2 summarize the methylation levels at DM loci, including their associated genes, q-values, partial correlation coefficients, and corresponding *p*-values.

## 3. Discussion

This pilot study is the first to identify a correlation between DNA methylation patterns at birth and the neurodevelopmental trajectories of children exposed to maternal GDM, establishing a hypothesis-generating foundation for this field. We observed significant declines in cognitive, language, and motor scores among exposed offspring compared to unexposed controls. Notably, the identification of 28 DMR-associated genes whose methylation status directly correlates with Bayley-III performance in GDM-exposed infants highlights a specific epigenetic link between the intrauterine environment and childhood brain development.

The synthesis of results from the various enrichment databases reveals a convergent biological profile: the majority of GDM-associated DMRs are located in genes fundamental to the structural and functional architecture of the central nervous system. The convergence of pathways related to neurogenesis (GO) [13], synaptic membrane components (COMPARTMENTS) [14] and axonal guidance (SynGO) [14] suggest a coordinated epigenetic response to the diabetic intrauterine environment. This cross-category consistency strengthens our hypothesis that GDM-induced methylation changes specifically target neurodevelopmental programming rather than being distributed across unrelated physiological systems. Notably, similar pathways were previously identified in a study of infants exposed to maternal T1DM [16], suggesting a shared epigenetic response.

Our prior research has documented neurodevelopmental abnormalities in the offspring of mothers with GDM and type 1 diabetes, including altered video-EEG patterns [17], difficulties in achieving stable behavioral states [18], and deficits in memory and learning pathways observed both in utero and during the neonatal period [19]. Maternal glycosylated hemoglobin levels have been directly linked to these developmental deficits [17,18,19]. The significantly higher birthweights and increased prevalence of large-for-gestational-age newborns observed in the GDM-exposed cohort serve as an objective manifestation of the hyperglycemic milieu experienced in utero. These findings align with established evidence regarding the stimulatory effect of maternal glucose levels on fetal adiposity and overgrowth [20], providing a clear clinical link between the intrauterine metabolic environment and neonatal anthropometric outcomes.

Recent research in rodent models [21] demonstrates that high glucose exposure alters DNA methylation and gene expression in neural progenitor cells, disrupting pathways essential for neurogenesis, axon guidance, and synaptic development. These findings provide a biological basis for neurodevelopmental impairment that aligns with our observations.

A meta-analysis of over 56 million mother–child pairs revealed that maternal diabetes—both pre-gestational and gestational—significantly elevates the risk of neurodevelopmental disorders, specifically affecting, among other aspects, communication, motor, and learning domains [4]. Consistent with these observations, infants in our cohort exposed to GDM exhibited significantly lower scores across these three neurodevelopmental domains at two years of age compared to controls. Various pathophysiological mechanisms have been suggested to mediate this impact, ranging from anemia, immune, and inflammatory disruptions to epigenetic reprogramming [22].

Research linking in utero DNA methylation to offspring neurodevelopmental outcomes remains scarce, and findings are inconsistent across cohorts. While one meta-analysis identified correlations only within the *INPP5A* gene [23], another reported associations between *SPTBN4* and severe developmental delay [24]. Conversely, Caramaschi et al. [25] found no significant association at all. In our study, *SPTBN4* and *INPP5B*—a member of the same inositol polyphosphate 5-phosphatase family as the previously reported *INPP5A*—also exhibited DMRs; however, methylation levels did not correlate with the children’s scores on the Bayley-III Scales.

More recently, Diez-Ahijado et al. (2024) [26] identified four CpGs associated with general cognitive scores, annotated to *MECOM*, *PSMG2*, *CEP76*, and *DAB2*. We also identified significant DMRs in *PSMG2* and *CEP76* as well as in *DAB1*—a member of the same family as *DAB2*. Notably, *DAB1* plays a significantly more prominent role in neurodevelopment than *DAB2*, and its prenatal expression has just been shown to be affected by maternal hyperglycemia [27].

Our findings further align with those reported by Howe et al. [28] in their investigation of the newborn methylome following GDM exposure, although their study did not evaluate neurodevelopmental outcomes. These authors identified significant hypomethylation across two specific regions associated with maternal GDM, the promoter of *OR2L13*—a locus previously implicated in autism spectrum disorder—and the gene body of *CYP2E1*, encoding an enzyme typically upregulated in both type 1 and type 2 diabetes. The promoter of *OR2L13* and the *CYP2E1* gene were also differentially methylated in our cohort as well as in González-González [16] involving infants born to mothers with pre-gestational type 1 diabetes mellitus.

Partial correlation analyses point toward a link between lower scores achieved on the Bayley-III Scales by the offspring of mothers with GDM and DNA methylation signatures at birth. The identified DMRs were located in genes putatively involved in the structural and functional assembly of the central nervous system and glucose metabolism (Table 6). However, given the exploratory nature of this pilot study and the limited sample size, these associations should be interpreted as hypothesis-generating rather than definitive evidence of a causal mechanism.

In particular, our study highlighted a significant cluster of genes associated with brain scaffolding and maturation. Notably, the correlation observed between methylation levels in the DMR-associated *WNT4*—a gene known for its role in synapse formation and neuronal circuitry [29]—and all Bayley-III domains provides a compelling signal. Nevertheless, it is important to note that the presence of a DMR does not inherently imply a functional alteration in gene expression. Additionally, we identified a significant cluster of genes involved in the scaffolding and maturation of the brain, such as *PCDHG* alfa and beta clusters, which act as molecular barcodes that provide neurons with a unique identity and the precise refinement of synaptic connections [30,31], and *TBCD* and *PALM*, essential for the dynamics of the neuronal cytoskeleton [32,33]. The identification of DMRs in genes such as *TP73-AS1* [34], *PDE9A* [35], and *NLRC5* [36]—all previously implicated in hippocampal maturation and memory functions—provides a plausible, though exploratory, biological basis for the neurodevelopmental variations observed in the Bayley-III Scales among children exposed to maternal GDM. The altered methylation patterns in *FOXF2* and *CHFR* raise the tentative possibility that maternal GDM might influence the development of the blood–brain barrier (BBB) [37,38,39]. The fact that *FOXF2* correlated with all Bayley-III domains and *CHFR* correlated with two marks this pathway as a priority for further investigation. A compromised BBB during critical windows of development could increase the brain’s vulnerability to systemic metabolic insults, further compounding the risk of neurodevelopmental delays. Furthermore, the presence of DMRs in genes like *HOXB3* and *CSNK1D* [40,41] suggests a direction for future research into whether GDM-induced epigenetic programming might have long-term implications for neurobiological aging. Finally, the metabolic and hormonal regulation category underscores the systemic nature of GDM-induced programming. The correlation of Bayley-III scores with methylation levels in DMR-associated genes such as *FSTL3* and *H6PD* [42,43,44,45] highlights a sophisticated “cross-talk” between peripheral metabolic health and neurocognitive development. These genes, traditionally associated with insulin resistance and placental growth, appear to be integrated into the broader neurodevelopmental outcome of the child. Of particular interest is the novel association of *LOC101928978* with cognitive and language domains. While previously uncharacterized in this context, its significant correlation in our pilot study marks it as a potential candidate for future functional validation.

Collectively, these epigenetic signatures suggest that GDM does not merely impose a transient metabolic stress but induces a stable reprogramming of genes essential for the structural and functional assembly of the central nervous system.

Monitoring these possible epigenetic markers in neonates exposed to maternal diabetes could facilitate the early identification of children at risk for cognitive or motor delays. Such a proactive approach would allow for the implementation of targeted neuro-rehabilitative interventions during the critical windows of brain plasticity in early childhood, ultimately mitigating the long-term impact of maternal hyperglycemia on offspring health.

**Table 6:** Functional category of genes containing DMRs associated with offspring neurodevelopment Bayley-III domains.

Functional Category	DMR-Associated Genes	Biological Function	Reference
Structural and functional assembly of the CNS	*PCDHGA1-11*, *PCDHGB1-8P* *PCDH* gamma and beta clusters	Neurodevelopmental regulators, neuronal survival and synaptic signaling hubs	(Hanes et al., 2026) [30] (May et al., 2025) [31]
	*TBCD*	Cerebral cortex development, dynamics and behavior of the neuronal cytoskeleton	(Edvardson et al., 2016) [32]
	*TMEM132D*	Neural circuit maturation and putative myelination	(Hjoj & Bozzi, 2025) [46]
	*KCNT1*	Regulation of neuronal excitability, circuit maturation, and interneuron differentiation	(Wei et al., 2026) [47]
	*PALM*, *BTBD6*	Cytoskeleton of neurons; maturation of late neuronal markers and axon organization	(Macarrón-Palacios et al., 2025) [33]; (Bury et al., 2008) [48]
	*BRF1*	Modulation of neuronal cell-surface signaling	(Borck et al., 2015) [49]
	*GALNT9*	Maintains mitochondrial integrity in dopaminergic neurons	(Peng et al., 2024) [50]
	*TP73-AS1*	Essential for hippocampal development	(Amelio et al., 2020) [34]
	*MEST*	Recognition memory, social behavior or maternal behavior	(Anunciado-Koza et al., 2022) [51]
	*PDE9A*	Synaptic plasticity, memory and cognition	(Rosenbrock et al., 2019) [35] (Kleiman et al., 2012) [52]
	*FMO2*	Neurotransmitter regulator	(Zhang et al., 2026) [53]
	*HOXB3*	Potential biomarker for early diagnosis of Alzheimer’s disease	(Kreicberga et al., 2021) [40]
	*WNT4*	Synapse formation and neuronal circuitry	(Salinas, 2012) [29]
	*CSNK1D*	Circadian rhythms regulator; neurodegenerative diseases	(Xu et al., 2019) [41] (Lee et al., 2011) [54]
	*NLRC5*	Essential for hippocampal development and synapse plasticity	(Li et al., 2020) [36]
	*FOXF2*, *CHFR*	Development and maintenance of the blood–brain barrier	(Reyahi et al., 2015) [37] (Xie et al., 2026) [38] (Wu et al., 2021) [39]
Metabolic and Hormonal Regulation	*FSTL3*	Reduced in GDM-affected placentas; body composition and insulin resistance; cognition and dementia	(Hu et al., 2012) [42]; (Brown et al., 2011) [43]; (Newman et al., 2023) [44]
	*H6PD*, *C1QTNF6*	Insulin resistance, metabolic syndrome and type 2 diabetes	(Bánhegyi et al., 2009) [45]; (Lei et al., 2017) [55]
	*C1QTNF8*, *SLC37A1*, *PPP2R5C*, *GNAS*	Metabolism and immunity; glucose–phosphate antiporter; placental and fetal growth	(Schäffler & Buechler, 2012) [56]; (Pan et al., 2011) [57]; (Luo et al., 2026) [58]; (McMinn et al., 2006) [59]
Novel Association	*LOC101928978*	Significantly associated with cognitive and language domains	This study

However, it is important to acknowledge that the neurodevelopmental differences observed in the GDM-exposed group, specifically the lower scores in cognitive, language, and motor domains, likely reflect a complex interplay of clinical factors beyond glucose intolerance alone. In our cohort, maternal pre-pregnancy BMI and age differed significantly between groups, and both variables are recognized factors that may influence infant development. While these baseline differences make it challenging to isolate GDM as the sole driver of the Bayley-III outcomes, our findings suggest a potential mechanistic link through DNA methylation. Notably, the 1053 DMRs identified in this study were adjusted for maternal BMI and age during the discovery phase, which supports the hypothesis that this epigenetic signature is associated with the diabetic intrauterine environment. Nevertheless, these correlations should be interpreted with caution, as they likely represent the integrated impact of a suboptimal metabolic milieu where GDM and maternal adiposity may exert synergistic effects on the developing brain.

A primary strength of this research lies in its pioneering nature, as it represents the first prospective, hypothesis-generating study to link neonatal DNA methylation in offspring exposed to maternal GDM to subsequent neurodevelopmental trajectories. A significant methodological advantage was the application of the TruSeq-Methyl-Capture-EPIC platform. By capturing over 3.3 million CpG sites per sample, our approach provided a comprehensive and high-resolution characterization of the methylome, far exceeding the resolution of traditional platforms such as the Illumina 450K or EPIC arrays, which are limited to approximately 450,000–850,000 CpG loci. This expanded genomic coverage accounts for the higher number of DM CpGs and DMR-associated genes identified in our cohort compared to the existing literature.

The main weakness of this research lies in its limited sample size, which necessitates an exploratory interpretation of the findings. Nevertheless, to ensure the reliability of our results, a power analysis was conducted considering the complexity of the methylome. By prioritizing the identification of DMRs to reduce dimensionality to approximately 5000–10,000 biologically informative regions, our sample size (n ≈ 27 per group) provides 80% power to detect a mean methylation difference of 5% (assuming a standard deviation of 5% and a large effect size, Cohen’s d = 1.0) at a 95% confidence level. This strategy confirms that this study is sufficiently powered to identify prominent epigenetic signals associated with GDM-induced neurodevelopmental changes. Although the regression analysis accounted for maternal age, pre-gestational BMI, newborn sex, and gestational age at delivery, further adjustment for variables such as cord blood cell-type heterogeneity, maternal glycemic levels, and socioeconomic indicators was omitted to avoid over-parameterization, as dictated by the limited cohort size. Finally, without concurrent expression data, the biological impact of the identified DMRs should be viewed as hypothesis-generating and warrants further functional validation. Consequently, rather than offering conclusive results, this study establishes a hypothesis-generating framework that identifies novel epigenetic targets for future, more robustly powered studies within a previously unaddressed clinical context.

Another limitation of this study is the use of cord blood as a surrogate for central nervous system tissue. While DNA methylation is tissue-specific, evidence suggests that inter-individual variation in certain genomic regions is highly correlated between blood and brain [23,60]. Thus, our results should be viewed as systemic epigenetic markers of the intrauterine environment that reflect, even if indirectly, the programming of neurodevelopmental trajectories.

In conclusion, while these preliminary and exploratory findings require further validation in larger cohorts, this study provides pioneering evidence that neurodevelopmental alterations identified in children born to mothers with GDM are associated with intrauterine DNA methylation changes detectable in umbilical cord blood at birth.

## 4. Material and Methods

This prospective, hypothesis-generating pilot cohort study included 60 mother–offspring pairs. The participants were divided into two groups: infants born to mothers with gestational diabetes mellitus (GDM group, n = 30) and a control group of infants born to mothers with normal glucose tolerance during pregnancy (Control group, n = 30). Recruitment was conducted at the University Hospital of the Canary Islands (Tenerife, Spain) between June 2021 and January 2024.

GDM was diagnosed, and normal glucose tolerance was assured, using a two-step approach. Initially, a screening test (O’Sullivan test) was performed; if positive, the diagnosis was confirmed via a 100 g oral glucose tolerance test (OGTT). Following the recommendations of the Spanish Group for Diabetes and Pregnancy (GEDE) and the National Diabetes Data Group (NDDG) criteria [61], GDM was confirmed when at least two values met or exceeded the following thresholds: fasting glucose, 105 mg/dL; 1 h, 190 mg/dL; 2 h, 165 mg/dL; and 3 h, 145 mg/dL. Glycosylated hemoglobin (HbA1c) levels were monitored periodically throughout the pregnancy.

Inclusion criteria were singleton pregnancy, gestational age > 38 weeks, no pre-existing maternal pathology, no history of smoking or drug abuse, and no pharmacological treatment during pregnancy (with the exception of insulin in the GDM group), mode of delivery (vaginal or elective cesarean section under regional anesthesia) and absence of perinatal complications. The GDM group comprised pregnant women diagnosed during the second trimester of gestation (24–28 weeks). This specific timeframe was selected to exclude participants with undiagnosed pre-gestational glucose intolerance, thereby minimizing the potential confounding effects of hyperglycemia during early pregnancy on embryonic and fetal development.

Exclusion criteria included a newborn birthweight below the 10th or above the 90th percentile in the Control group, according to customized growth curves for Spanish children [62]. Additionally, participants from both groups were excluded if the infant presented any significant neonatal or childhood pathology during the follow-up period, with the exception of common, age-appropriate pediatric illnesses.

This study was conducted in accordance with the Declaration of Helsinki and was approved by the Clinical Ethics Committee of the Canary Islands University Hospital Complex, Spain (CHUC-201817, approved on 28 March 2019), and written informed consent was obtained from all mothers prior to participation.

### 4.1. Collection and Storage of Samples and DNA Purification

Cord blood was collected immediately after birth in 3 mL K3-EDTA tubes (BD Vacutainer, Franklin Lakes, NJ, USA) in the delivery room and immediately stored at −80 °C until DNA extraction. DNA of cord blood samples was purified using the Blood genomicPrep Mini Spin Kit (Cytiva Amersham™, Marlborough, MA, USA). DNA concentrations were measured on the Qubit 3.0 fluorometer using the Qubit dsDNA HS Assay (Thermo Fisher Scientific, Waltham, MA, USA).

### 4.2. Bisulfite Conversion and Methyl-Seq Procedures

Bisulfite conversion of DNA and library preparation were done using the TruSeq-Methyl-Capture-EPIC Library Prep Kit (Illumina Inc., San Diego, CA, USA) [63], which targets 3.34 million CpG sites, following the manufacturer’s recommendations. Library sizes were assessed with a 4200 TapeStation (Agilent Technologies, Santa Clara, CA, USA), and the concentration was determined with the Qubit dsDNA HS Assay (Thermo Fisher Scientific, Waltham, MA, USA). Sequencing was conducted at the Instituto Tecnológico y de Energías Removables (ITER, Santa Cruz de Tenerife, Canary Islands, Spain) with a NovaSeq 6000 Sequencing System (Illumina Inc.) using 110 bp paired-end single-indexed reads along with 1% of a PhiX Control-V3 (Illumina Inc.) [63].

### 4.3. Bioinformatics Analysis of Sequences

We used bcl2fastq v2.20 [64] to perform sample demultiplexing and Bismark v0.24.0 [65] for bisulfite mapping and methylation calling against the GRCh37/hg19 reference genome. The Bismark-based pipeline transformed the sequence and reads into fully bisulfite-converted forward (C→T) and reverse read (G→A) conversion of the forward strand versions. Then, transformed reads were aligned to similarly converted versions of the reference genome (also C→T and G→A converted). The best alignment from the four alignment processes against the bisulfite genomes was compared to the normal genomic sequence, and the methylation state of all cytosine positions in the read was inferred.

### 4.4. Sequencing Quality Control and Data Processing

To ensure the reliability of the DNA methylation data, several quality control (QC) metrics were monitored throughout the workflow. Sequencing was performed to achieve an average depth of >30× per targeted region, with a mean mapping efficiency of approximately 72% using Bismark, which is consistent with the expected performance of the TruSeq-Methyl-Capture-EPIC kit. Bisulfite conversion efficiency was strictly evaluated by calculating the conversion rate of non-CpG cytosines, with all samples included in the final analysis exceeding 99% efficiency.

Data filtering was performed at the CpG level, retaining only sites with at least 10× coverage across all samples to ensure statistical robustness in the identification of DMRs. To minimize potential technical bias, samples from the GDM and Control groups were randomized during library preparation and sequencing runs. Furthermore, principal component analysis (PCA) was conducted on the final methylation dataset; the absence of clustering by processing batch confirmed that the primary source of variance was the biological group (GDM vs. Control) rather than technical artifacts.

### 4.5. Bioinformatics and Statistical Analysis

**Figure 3:**
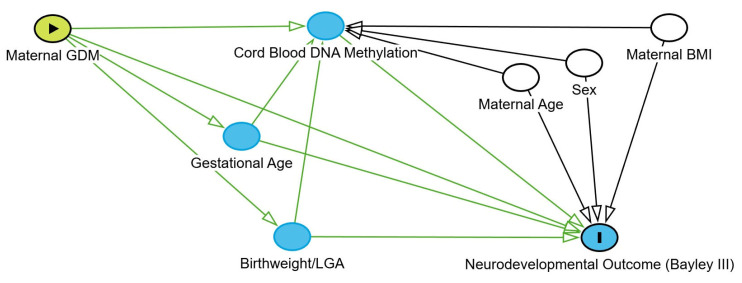
Directed acyclic graph (DAG) of the structural causal model. This diagram delineates the hypothesized pathways through which maternal GDM influences neurodevelopmental outcomes at age two years. The model incorporates cord blood DNA methylation, gestational age, and birthweight/LGA as intermediate variables (mediators) within the causal chain. Maternal age, pre-pregnancy BMI, and infant sex are represented as potential confounders. Additionally, birthweight/LGA is utilized as a covariate in the partial correlation analyses to account for fetal growth while isolating the independent effect of DNA methylation. Green arrows indicate the hypothesized direction of biological influence. Regarding the nodes, the green circle with a triangle represents the exposure variable (Maternal GDM), the blue circle with an ‘I’ indicates the outcome variable (Neurodevelopmental Outcome), and the plain blue circles represent intermediate mediators. White circles denote adjusted covariates or potential confounders.

Methylation analysis was conducted using the MethylKit R package (version 1.32.1) [66]. After loading the methylation data for each individual, a filter based on read coverage was applied to reduce bias associated with enrichment. Bases with coverage below 10× and above the 99.9th percentile of coverage in each sample were discarded. For subsequent analyses, all samples were combined into a single object for base pair locations covered in all samples. The calculate DiffMeth function was used to identify CpG sites or regions with methylation differences using logistic regression. To clarify the hypothesized causal structure and justify the selection of covariates, a directed acyclic graph (DAG) was developed [67] (Figure 3). Based on this framework, maternal age, infant sex, and pre-pregnancy BMI were treated as confounders and included in the models. Gestational age was included as a covariate in the partial correlation analysis to adjust for tissue maturity and isolate the direct effect of maternal GDM on the methylome. Conversely, birthweight and LGA status were identified as downstream mediators and were excluded from the primary models to avoid overadjustment and collider bias.

*p*-values were adjusted to q-values to control the false discovery rate (FDR) in multiple hypothesis testing using the sliding linear model (SLIM). The MethSeg function was applied to create CpG segments with similar profiles of differential methylation. Genomation R package (version 1.38.0) and data obtained from the Hg19 known Genea BED-file genome browser website [68] were used for gene location and promoters, introns, exons, and intergenic region identification.

Differentially methylated (DM) CpGs and DM regions (DMRs, genomic clusters of ≥3 DM-CpGs and ≤1000 base pairs) were identified. CpG sites were considered DM if they showed a methylation difference > 15% and a q-value < 0.01. For DMRs, the threshold was set at a mean methylation difference ≥ 1% and a q-value < 0.01. Positive and negative methylation differences coefficients refer to hypermethylation and hypomethylation, respectively. DMR-associated genes were defined as those whose genomic coordinates overlap with one or more DMRs.

### 4.6. Enrichment Analysis and Functional Characterization

An initial broad functional assessment of DMR-associated genes was conducted using the Gene Ontology (GO) database [13]. To further assess their involvement in neurodevelopment and brain functions, the COMPARTMENT Curated 2025 and SynGO 2024 databases, within the Enrichr platform [14], were utilized. Pathways with a false discovery rate (FDR)-adjusted *p*-value (q-value) less than 0.05 were considered statistically significant.

### 4.7. Neurodevelopment Assessment

At 24–26 months of age, children underwent neurodevelopmental assessment via the Bayley Scales of Infant Development-Third Edition (Bayley-III) [15]. Two psychometricians, blinded to the clinical groups (GDM vs. Control), performed all evaluations.

The relationship between DMR methylation levels and the various neurodevelopmental domains of the Bayley-III Scales was evaluated using partial correlation analysis. To account for the potential confounding effect of gestational age at delivery on both the epigenome and developmental outcomes, partial correlations adjusted for this variable were employed. To minimize the risk of false positives while maintaining sufficient power in this pilot cohort, a more stringent significance pre-specified threshold of *p* < 0.005 was adopted for the correlation analyses.

## Figures and Tables

**Table 1:** Maternal characteristics, perinatal outcomes and Bayley-III Scale domain scores.

	Control Group (n = 30)	GDM Group (n = 30)	*p*-Value
Maternal characteristics			
Age (years)	31.5 ± 5.1	34.1 ± 5.1	0.047
Pre-pregnancy weight (kg)	64.2 ± 17.8	93.0 ± 15.7	<0.001
Height (cm)	162.0 ± 5.3	162.6 ± 6.5	0.332
Body mass index (kg/m^2^)	24.4 ± 6.2	35.2 ± 6.1	<0.001
Parity = 0, n (%)	25 (83)	12 (40)	0.001
Perinatal outcomes			
Cesarean, n (%)	6 (20)	7 (23)	0.754
Gestational age (days)	275.8 ± 4.9	272.7 ± 8.0	0.071
Newborn weight (g)	3317 ± 298	3724 ± 606	0.002
Weight percentile (%)	60.5 ± 23.2	72.5 ± 32.1	0.104
LGA n (%)	2 (7)	15 (50)	<0.001
Sex = Female, n (%)	17 (57)	16 (53)	0.795
Apgar test at 1st min	8.9 ± 0.7	8.9 ± 0.7	0.981
Apgar test at 5 min	9.0 ± 0.6	9.0 ± 0.3	0.993
Bayley-III Scales	(n = 28)	(n = 27)	
Cognitive (%)	116.7 ± 13.8	98.7 ± 14.0	<0.001
Language (%)	114.8 ± 15.0	94.9 ± 20.6	<0.001
Motor (%)	111.7 ± 10.6	98.3 ± 13.6	<0.001
Score < 95%, n (%)			
Cognitive	1 (4)	6 (22)	0.051
Language	3 (11)	12 (44)	<0.001
Motor	1 (4)	10 (37)	0.002

Results are shown as percentages or mean and standard deviation. LGA, large for gestational age. Data normality was assessed by inspecting the frequency distributions and the Kolmogorov–Smirnov test. Differences were studied using Student’s *t*-test. Comparison between proportions was performed using the chi-squared test and the Fisher’s exact test with small sample sizes (<5).

**Table 2:** Gene Ontology enrichment analysis [13] of DMR-associated genes identified in newborns exposed to GDM: neurodevelopmental and neurological function pathways.

		Reference List		Upload			
Uniquely Mapped IDS:		20,580 out of 20,580		860 out of 885			
Unmapped IDs:		0		192			
Multiple mapping information:		0		27			
	Homo sapiens						
	(References)						
GO biological process complete	#	##	expected	Fold Enrichment	+/−	raw *p*-value	FDR
Vocal learning	8	4	0.34	11.63	+	2.07 × 10^−4^	3.02 × 10^−2^
imitative learning	8	4	0.34	11.63	+	2.07 × 10^−4^	3.05 × 10^−2^
observational learning	9	4	0.39	10.34	+	3.60 × 10^−4^	4.32 × 10^−2^
learning	164	21	7.05	2.98	+	7.84 × 10^−6^	2.76 × 10^−3^
learning or memory	288	26	12.38	2.1	+	3.54 × 10^−4^	4.32 × 10^−2^
behavior	664	53	28.55	1.86	+	1.50 × 10^−5^	4.53 × 10^−3^
Walking behavior	40	8	1.72	4.65	+	2.56 × 10^−4^	3.53 × 10^−2^
Axon guidance	233	25	10.02	2.5	+	2.58 × 10^−5^	6.57 × 10^−3^
Neuron projection guidance	234	25	10.06	2.48	+	2.78 × 10^−5^	6.94 × 10^−3^
neuron projection development	729	58	31.35	1.85	+	6.42 × 10^−6^	2.63 × 10^−3^
neuron development	916	74	39.39	1.88	+	1.69 × 10^−7^	1.32 × 10^−4^
neuron differentiation	1156	83	49.71	1.67	+	4.43 × 10^−6^	2.04 × 10^−3^
generation of neurons	1235	91	53.11	1.71	+	4.67 × 10^−7^	3.13 × 10^−4^
neurogenesis	1433	104	61.62	1.69	+	1.14 × 10^−7^	1.12 × 10^−4^
neuron projection morphogenesis	508	43	21.85	1.97	+	3.05 × 10^−5^	7.51 × 10^−3^
Axonogenesis	383	36	16.47	2.19	+	1.08 × 10^−5^	3.63 × 10^−3^
cell morphogenesis involved in neuron differentiation	465	40	20	2	+	3.74 × 10^−5^	8.49 × 10^−3^
axon development	439	39	18.88	2.07	+	2.15 × 10^−5^	5.76 × 10^−3^
Central nervous system development	1057	75	45.45	1.65	+	2.25 × 10^−5^	5.93 × 10^−3^

Results for FDR, *p* < 0.05. # Number of genes in the reference list (Homo sapiens). ## Number of genes form the analyzed dataset.

**Table 3:** COMPARTMENTS Curated 2025 enrichment analysis [14] of DMR-associated genes identified in newborns exposed to GDM.

Index	Name	*p*-Value	Adjusted *p*-Value	Odds Ratio	Combined Score
1	Main axon	0.000061	0.02744	5.5	53.4
2	Cell projection membrane	0.000091	0.02744	2.46	22.91
3	Axon initial segment	0.00036	0.07236	11.31	89.65
4	Brush border membrane	0.001156	0.1742	4.39	29.71
5	Ciliary membrane	0.002131	0.2104	3.55	21.81
6	Cation channel complex	0.002226	0.2104	2.08	12.68
7	Ion channel complex	0.002443	0.2104	1.99	11.95
8	Cytoplasmic side of plasma membrane	0.004336	0.3268	2.24	12.16
9	Brush border	0.006062	0.3664	2.96	15.13
10	Neuron projection	0.006076	0.3664	1.44	7.37

**Table 4:** SynGO (2024) enrichment analysis [14] of genes associated with differentially methylated regions (DMRs) in newborns exposed to maternal gestational diabetes.

Index	Name	*p*-Value	Adjusted *p*-Value	Odds Ratio	Combined Score
1	Regulation of postsynaptic neurotransmitter receptor activity	0.0007524	0.04407	6.79	48.82
2	Postsynaptic density intracellular component	0.001062	0.04407	3.98	27.25
3	Integral component of presynaptic membrane	0.003226	0.08924	2.39	13.71
4	Postsynapse	0.01098	0.2278	1.76	7.93
5	Regulation of postsynapse organization	0.02238	0.2576	2.69	10.24
6	Synaptic vesicle endocytosis	0.02410	0.2576	2.93	10.92
7	Integral component of postsynaptic membrane	0.02416	0.2576	2.01	7.49
8	Regulation of postsynaptic neurotransmitter	0.02485	0.2576	12.03	44.44
9	Structural constituent of postsynaptic density	0.02793	0.2576	5.42	19.38
10	Modulation of chemical synaptic transmission	0.03533	0.2716	2.03	6.8

**Table 5:** Associations between DMR methylation levels and Bayley-III neurodevelopmental scores. The table presents differentially methylated regions (DMRs) annotated to specific genes, their methylation differences between the GDM and Control groups, and false discovery rate (FDR) q-values. Correlation coefficients (r) and *p*-values are provided for partial correlation (adjusting for gestational age) across cognitive, language, and motor domains. Significant associations (pre-specified exploratory threshold, *p* < 0.005) are highlighted in pink.

Gene	DM Region	Diff. Methylation (Cases-Control)	q-Value	Partial Correlation (Controlling by Gestational Age)		
				Domain		
				Cognitive	Language	Motor
*TBCD*	chr17: 80735979–80817580	5.956455	<0.000001	0.472 (<0.001)	0.392 (0.003)	0.274 (0.045)
*C1QTNF6*	chr22:37578579–37584441	−7.810497	<0.000001	0.423 (0.001)	0.337 (0.013)	0.235 (0.088)
*FSTL3/PALM*	chr19:679162–709809	−4.658835	0.001509	−0.420 (0.002)	−0.211 (0.126)	−0.222 (0.106)
*FOXF2*	chr6:1394714–1395164	−3.998630	0.000175	0.410 (0.002)	0.383 (0.004)	0.508 (<0.001)
*WNT4*	chr1:22456024–22456168	3.007232	<0.000001	−0.398 (0.003)	−0.372 (0.005)	−0.406 (0.002)
*H6PD*	chr1:9330474–9338423	4.349031	<0.000001	−0.396 (0.003)	−0.305 (0.025)	−0.228 (0.098)
*CHFR*	chr12:133412917–133413127	−3.289805	<0.000001	0.390 (0.004)	0.398 (0.003)	0.326 (0.016)
*PCDHGA1-8*; *A11*; *B1-4*; *B8P*	chr5:140805681–140807423	−4.894645	<0.000001	0.382 (0.004)	0.272 (0.046)	0.298 (0.029)
*LOC101928978*	chr4:85220283–85372711	−3.935369	<0.000001	0.378 (0.005)	0.397 (0.003)	0.366 (0.007)
*NLRC5*	chr16:57034712–57041096	−3.352412	0.000023	0.236 (0.086)	0.424 (0.001)	0.289 (0.034)
*C1QTNF8*	chr22:1138479–1139936	−4.823317	<0.000001	0.266 (0.052)	0.416 (0.002)	0.335 (0.013)
*PPP2R5C*	chr14:102312543–102316169	−6.355594	<0.000001	0.301 (0.027)	0.406 (0.002)	0.301 (0.027
*KCNT1*	chr9:138660908–138663444	−3.333032	<0.000001	0.259 (0.058)	0.404 (0.002)	0.250 (0.069)
*SLC37A1/PDE9A*	chr21:43954849–44104950	−1.985161	<0.000001	0.245 (0.074)	0.398 (0.003)	0.178 (0.197)
*CSNK1D*	chr17:80200667–80200785	4.244254	<0.000001	−0.242 (0.078)	−0.395 (0.003)	−0.160 (0.249)
*TP73-AS1*	chr1:3659742–3663114	−2.834900	0.000059	0.136 (0.325)	0.386 (0.004)	0.154 (0.266)
*ENPP7P13*	chr16:33573660–33573849	4.348636	0.000054	−0.214 (0.121)	−0.307 (0.024)	−0.492 (<0.001)
*GALNT9*	chr12:132859414–132859824	−4.116222	0.000198	0.239 (0.081)	0.247 (0.072)	0.429 (0.001)
*TMEM132D*	chr12:130347493–130357315	−4.985490	0.000015	0.275 (0.044)	0.219 (0.112)	0.399 (0.003)
*MEST*	chr7:130131189–130131519	6.927757	0.000011	−0.038 (0.785)	−0.202 (0.144)	−0.397 (0.003)
*BRF1/BTBD6*	chr14:105715658–105715970	−2.020851	0.001288	0.114 (0.413)	0.126 (0.362)	0.388 (0.004)
*FMO2*	chr1:171176736–171194570	−4.636900	<0.000001	0.206 (0.135)	0.295 (0.030)	0.383 (0.004)
*SCGB1B2P*	chr19:35064490–35068221	−3.460115	<0.000001	0.097 (0.484)	0.280 (0.041)	0.381 (0.004)
*LINC01626*	chr6:72130500–72130905	3.200324	0.000134	−0.114 (0.411)	−0.330 (0.015)	−0.381 (0.004)
*HOXB3*	chr17:46656695–46658976	−3.376877	0.000078	0.309 (0.023)	0.335 (0.014)	0.380 (0.005)
*GNAS*	chr20:57416606–57417048	5.822824	<0.000001	−0.290 (0.033)	−0.218 (0.113)	−0.378 (0.005)

## Data Availability

The data presented in this study are available on reasonable request from the corresponding author.

## Supplementary Materials

The following supporting information can be downloaded at: https://www.mdpi.com/article/10.3390/ijms27083571/s1.
