# Supplementary material for: Association Between Maternal Gestational Diabetes, Cord Blood DNA Methylation, and Offspring Neurodevelopment
Source: Int J Mol Sci. 2026 Apr 16;27(8):3571. doi: 10.3390/ijms27083571 (PMC13115854; doi:10.3390/ijms27083571)
Supplement: Supplementary file 1 [file ijms-27-03571-s001.zip › ijms-4199067-supplementary.pdf]

Table S1.- Gene Ontology (GO) enrichment analysis

Results for **FDR p < 0.05**

	Reference list	upload_1
Uniquely Mapped IDS:	20580 out of 20580	860 out of 885
Unmapped IDs:	0	192
Multiple mapping information:	0	27

	Homo sapiens (REF)	upload_1(Hierarchy)					
**GO biological process complete**	#	#	expected	Fold Enrichment	+/-	raw P value	FDR
**1. Membrane depolarization during AV node cell action potential**	5	4	.22	18.60	+	1.64E-05	4.84E-03
AV node cell action potential	7	4	.30	13.29	+	1.07E-04	1.82E-02
AV node cell to bundle of His cell signaling	7	4	.30	13.29	+	1.07E-04	1.84E-02
regulation of multicellular organismal process	2996	168	128.84	1.30	+	2.50E-04	3.48E-02
cellular process	15556	718	668.95	1.07	+	6.22E-05	1.22E-02
regulation of biological quality	3033	185	130.43	1.42	+	4.21E-07	2.96E-04
**2. Regulation of arachidonate secretion**	6	4	.26	15.50	+	4.75E-05	1.00E-02
regulation of transport	1605	103	69.02	1.49	+	3.82E-05	8.40E-03
regulation of localization	2019	121	86.82	1.39	+	1.65E-04	2.54E-02
**3. Vocal learning**	8	4	.34	11.63	+	2.07E-04	3.02E-02
imitative learning	8	4	.34	11.63	+	2.07E-04	3.05E-02
observational learning	9	4	.39	10.34	+	3.60E-04	4.32E-02
learning	164	21	7.05	2.98	+	7.84E-06	2.76E-03
learning or memory	288	26	12.38	2.10	+	3.54E-04	4.32E-02
behavior	664	53	28.55	1.86	+	1.50E-05	4.53E-03
multicellular organismal process	6486	366	278.92	1.31	+	3.52E-10	7.42E-07
cognition	337	31	14.49	2.14	+	6.66E-05	1.28E-02
**4. Regulation of testosterone biosynthetic process**	9	4	.39	10.34	+	3.60E-04	4.35E-02
regulation of ketone biosynthetic process	23	6	.99	6.07	+	3.34E-04	4.18E-02
vascular endothelial cell response to laminar fluid shear stress	22	6	.95	6.34	+	2.56E-04	3.50E-02
regulation of platelet aggregation	31	8	1.33	6.00	+	3.70E-05	8.53E-03
regulation of platelet activation	55	10	2.37	4.23	+	1.03E-04	1.80E-02
regulation of homotypic cell-cell adhesion	40	9	1.72	5.23	+	3.97E-05	8.61E-03

regulation of cell adhesion	802	60	34.49	1.74	+	3.67E-05	8.60E-03
**5. Positive regulation of cartilage development**	33	7	1.42	4.93	+	4.25E-04	4.87E-02
positive regulation of developmental process	1337	88	57.49	1.53	+	6.47E-05	1.26E-02
regulation of developmental process	2475	142	106.43	1.33	+	3.16E-04	3.99E-02
positive regulation of multicellular organismal process	1678	110	72.16	1.52	+	7.49E-06	2.76E-03
**6. Walking behavior**	40	8	1.72	4.65	+	2.56E-04	3.53E-02
**7. Homophilic cell adhesion via plasma membrane adhesion molecules**	167	28	7.18	3.90	+	6.16E-10	1.14E-06
cell-cell adhesion via plasma-membrane adhesion molecules	261	34	11.22	3.03	+	8.71E-09	1.29E-05
cell-cell adhesion	551	56	23.69	2.36	+	2.86E-09	4.69E-06
cell adhesion	968	86	41.63	2.07	+	1.82E-10	4.48E-07
**8. Negative regulation of wound healing**	67	11	2.88	3.82	+	1.24E-04	2.03E-02
negative regulation of response to wounding	91	13	3.91	3.32	+	1.35E-04	2.15E-02
regulation of response to stimulus	4142	230	178.12	1.29	+	1.74E-05	5.04E-03
negative regulation of response to stimulus	1768	111	76.03	1.46	+	4.83E-05	1.00E-02
**9. Regulation of release of sequestered calcium ion into cytosol**	82	13	3.53	3.69	+	4.49E-05	9.61E-03
regulation of sequestering of calcium ion	133	17	5.72	2.97	+	5.74E-05	1.16E-02
regulation of calcium ion transmembrane transport	148	19	6.36	2.99	+	2.02E-05	5.52E-03
regulation of calcium ion transport	228	22	9.80	2.24	+	3.98E-04	4.67E-02
regulation of metal ion transport	355	31	15.27	2.03	+	1.80E-04	2.74E-02
regulation of monoatomic ion transport	425	36	18.28	1.97	+	1.37E-04	2.15E-02
regulation of monoatomic cation transmembrane transport	266	29	11.44	2.54	+	4.39E-06	2.09E-03
regulation of monoatomic ion transmembrane transport	295	29	12.69	2.29	+	3.38E-05	8.17E-03
regulation of transmembrane transport	412	34	17.72	1.92	+	3.03E-04	3.96E-02
**10. L-amino acid transport**	83	12	3.57	3.36	+	2.16E-04	3.13E-02
transport	3735	214	160.62	1.33	+	4.18E-06	2.05E-03
establishment of localization	3962	222	170.38	1.30	+	1.26E-05	4.05E-03
localization	4565	262	196.31	1.33	+	1.39E-07	1.21E-04
amino acid transport	137	17	5.89	2.89	+	8.36E-05	1.49E-02
**11. Positive regulation of B cell activation**	87	12	3.74	3.21	+	3.38E-04	4.19E-02
**12. Platelet activation**	102	14	4.39	3.19	+	1.18E-04	1.97E-02
blood coagulation	191	20	8.21	2.43	+	2.24E-04	3.14E-02
coagulation	193	20	8.30	2.41	+	2.57E-04	3.48E-02
regulation of body fluid levels	381	32	16.38	1.95	+	3.01E-04	3.97E-02

**13. Nerve development**	97	13	4.17	3.12	+	2.59E-04	3.48E-02
anatomical structure development	5370	335	230.93	1.45	+	4.62E-15	6.82E-11
developmental process	5903	344	253.85	1.36	+	3.14E-11	9.27E-08
nervous system development	2307	174	99.21	1.75	+	6.68E-14	2.47E-10
system development	3670	250	157.82	1.58	+	6.86E-15	5.06E-11
multicellular organism development	4074	267	175.19	1.52	+	5.72E-14	2.81E-10
inorganic cation import across plasma membrane	108	14	4.64	3.01	+	2.18E-04	3.09E-02
inorganic ion import across plasma membrane	108	14	4.64	3.01	+	2.18E-04	3.12E-02
inorganic ion transmembrane transport	782	62	33.63	1.84	+	3.52E-06	1.85E-03
transmembrane transport	1299	87	55.86	1.56	+	3.76E-05	8.40E-03
import across plasma membrane	169	19	7.27	2.61	+	1.26E-04	2.04E-02
import into cell	706	57	30.36	1.88	+	4.70E-06	2.10E-03
inorganic cation transmembrane transport	690	55	29.67	1.85	+	1.34E-05	4.20E-03
amino acid transmembrane transport	101	13	4.34	2.99	+	3.88E-04	4.58E-02
potassium ion transmembrane transport	168	21	7.22	2.91	+	1.14E-05	3.75E-03
potassium ion transport	187	23	8.04	2.86	+	5.84E-06	2.54E-03
metal ion transport	665	59	28.60	2.06	+	1.52E-07	1.24E-04
monoatomic cation transport	847	66	36.42	1.81	+	3.47E-06	1.90E-03
monoatomic ion transport	1037	76	44.59	1.70	+	6.18E-06	2.61E-03
monoatomic cation transmembrane transport	706	58	30.36	1.91	+	2.66E-06	1.57E-03
monoatomic ion transmembrane transport	864	66	37.15	1.78	+	6.63E-06	2.64E-03
**14. Protein localization to plasma membrane**	195	24	8.39	2.86	+	3.64E-06	1.85E-03
protein localization to cell periphery	239	26	10.28	2.53	+	1.40E-05	4.32E-03
protein localization to membrane	322	30	13.85	2.17	+	7.34E-05	1.39E-02
localization within membrane	562	45	24.17	1.86	+	7.60E-05	1.39E-02
cellular localization	2008	119	86.35	1.38	+	3.16E-04	4.02E-02
**15. Axon guidance**	233	25	10.02	2.50	+	2.58E-05	6.57E-03
**neuron projection guidance**	234	25	10.06	2.48	+	2.78E-05	6.94E-03
neuron projection development	729	58	31.35	1.85	+	6.42E-06	2.63E-03
neuron development	916	74	39.39	1.88	+	1.69E-07	1.32E-04
cell development	2351	157	101.10	1.55	+	1.56E-08	2.10E-05
cell differentiation	3806	216	163.67	1.32	+	7.45E-06	2.82E-03
cellular developmental process	3809	216	163.80	1.32	+	7.56E-06	2.72E-03
neuron differentiation	1156	83	49.71	1.67	+	4.43E-06	2.04E-03
generation of neurons	1235	91	53.11	1.71	+	4.67E-07	3.13E-04
neurogenesis	1433	104	61.62	1.69	+	1.14E-07	1.12E-04
plasma membrane bounded cell projection organization	1197	80	51.47	1.55	+	9.05E-05	1.59E-02
cell projection organization	1252	82	53.84	1.52	+	1.31E-04	2.10E-02

neuron projection morphogenesis	508	43	21.85	1.97	+	3.05E-05	7.51E-03
plasma membrane bounded cell projection morphogenesis	513	43	22.06	1.95	+	3.40E-05	8.09E-03
cell projection morphogenesis	520	44	22.36	1.97	+	2.30E-05	5.96E-03
anatomical structure morphogenesis	2303	154	99.04	1.55	+	1.67E-08	2.05E-05
cell morphogenesis	730	54	31.39	1.72	+	1.19E-04	1.98E-02
**axonogenesis**	383	36	16.47	2.19	+	1.08E-05	3.63E-03
cell morphogenesis involved in neuron differentiation	465	40	20.00	2.00	+	3.74E-05	8.49E-03
axon development	439	39	18.88	2.07	+	2.15E-05	5.76E-03
**16. Regulation of muscle system process**	246	24	10.58	2.27	+	1.85E-04	2.76E-02
chemical synaptic transmission	433	39	18.62	2.09	+	1.85E-05	5.24E-03
anterograde trans-synaptic signaling	433	39	18.62	2.09	+	1.85E-05	5.14E-03
trans-synaptic signaling	455	41	19.57	2.10	+	9.88E-06	3.39E-03
synaptic signaling	495	45	21.29	2.11	+	2.60E-06	1.60E-03
synapse organization	371	31	15.95	1.94	+	4.13E-04	4.80E-02
positive regulation of cell development	443	36	19.05	1.89	+	3.09E-04	3.96E-02
positive regulation of cell differentiation	870	66	37.41	1.76	+	7.38E-06	2.87E-03
regulation of cell differentiation	1577	97	67.82	1.43	+	3.61E-04	4.30E-02
brain development	761	54	32.73	1.65	+	3.42E-04	4.21E-02
animal organ development	2935	169	126.21	1.34	+	5.35E-05	1.10E-02
central nervous system development	1057	75	45.45	1.65	+	2.25E-05	5.93E-03
head development	812	58	34.92	1.66	+	1.84E-04	2.78E-02
**17. Cell migration**	958	67	41.20	1.63	+	8.30E-05	1.49E-02
**18. Negative regulation of signal transduction**	1423	93	61.19	1.52	+	5.87E-05	1.17E-02
regulation of signal transduction	3130	189	134.60	1.40	+	5.99E-07	3.84E-04
regulation of cell communication	3583	217	154.08	1.41	+	4.78E-08	5.43E-05
regulation of signaling	3579	215	153.91	1.40	+	1.03E-07	1.09E-04
negative regulation of cell communication	1518	97	65.28	1.49	+	7.57E-05	1.41E-02
negative regulation of cellular process	5145	268	221.25	1.21	+	2.99E-04	3.98E-02
negative regulation of signaling	1518	97	65.28	1.49	+	7.57E-05	1.40E-02
**19. Ribonucleoprotein complex biogenesis**	457	6	19.65	.31	-	4.16E-04	4.80E-02
**20. Detection of chemical stimulus involved in sensory perception of smell**	437	1	18.79	.05	-	1.37E-07	1.27E-04
detection of chemical stimulus involved in sensory perception	485	2	20.86	.10	-	1.90E-07	1.40E-04
detection of chemical stimulus	522	4	22.45	.18	-	2.86E-06	1.62E-03
sensory perception of smell	469	6	20.17	.30	-	3.06E-04	3.96E-02
