# Supplementary material for: Association Between Maternal Gestational Diabetes, Cord Blood DNA Methylation, and Offspring Neurodevelopment
Source: Int J Mol Sci. 2026 Apr 16;27(8):3571. doi: 10.3390/ijms27083571 (PMC13115854; doi:10.3390/ijms27083571)

Figure S1. Manhattan plot of terms from the COMPARTMENTS_Curated_2025 gene set [22]. Each point represents a single term along the x-axis. The y-values represent the $-\log_{10}$(p-value) corresponding to the enrichment of the input DMR-associated gene set for the term gene set. The top identified pathways associated with neurodevelopment or neurological functions are highlighted.

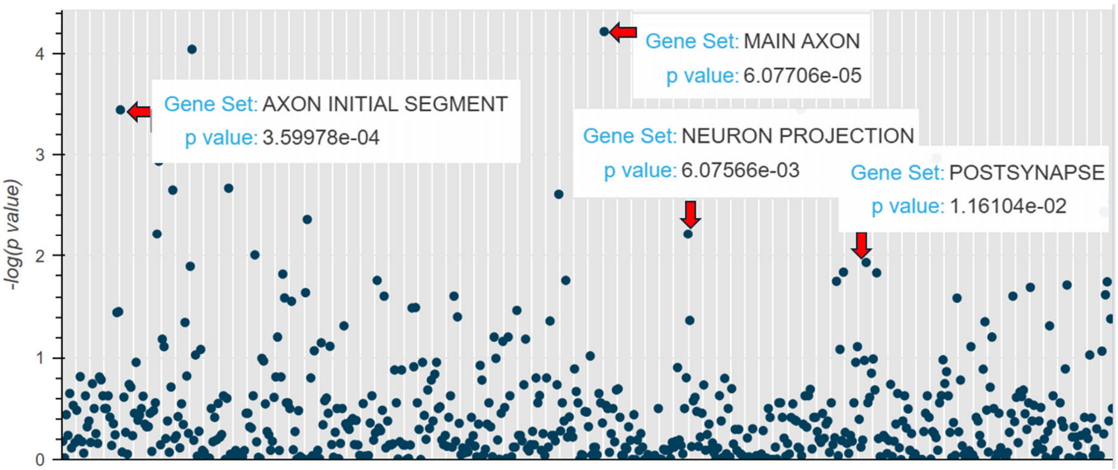

Supplement: Supplementary file 1 [file ijms-27-03571-s001.zip › Figure S1.(Supplementary material).pdf]
